# On Medical Reform

**Published:** 1810-02

**Authors:** 


### *To the Editors of the Medical and Physical Journal.*

GENTLEMEN,

IN your Journal of last month, (January, 1810) there is a letter addressed to you by a gentleman who signs himself OBSERVATOR, respecting the *hacknied custom,* as he is pleased to call it, of Physicians directing their prescriptions to be taken to the shop of a druggist to be prepared. I cannot but observe, that this letter deviates very widely from the *liberal principles* which he says many physicians are deficient in. After complaining of the practice of medical men, who direct their prescriptions to be made up by some particular druggist, he says, "for there are physicians who have their favorite houses'. And a little farther he adds, "it is not for me here to hazard an opinion as to the motives for so doing." Now, whatever may be this gentleman's secret opinion I know not; but I would ask him, what evil can possibly arise from this practice? If I be not mistaken, it is in many respects greatly advantageous. A man who is constantly in the habit of preparing the prescriptions of some one physician, becomes familiarly acquainted with his writing; therefore, as some physicians frequently write very unintelligible through haste, &c. this man is, doubtless, more capable of knowing the true meaning of his prescription than a stranger, and consequently less liable to error. This is not, I conceive, the only circumstance that ocasions medical men to make use of one particular shop. When a physician begins to practise in public, it is of essential importance that the medicines which he prescrtbes be genuine. In order to render this secure, he looks out for a man who, in his opinion, is qualified, and whose chemical and pharmaceutical preparations are in the highest perfection. This done, he fixes upon him as his compounder; and would it not be absurd for such to be continually changing from one shop to another?

I cannot imagine what Observator can adduce against what he terms, *physicians having their favoarite houses.* He seems to have a particular hostility against druggists, and in his haste to point out their defects, appears unconscious that his own arguments may be applied with equal force against himself. This passage of an ancient author, "Suo sibi gladio hunc jugulo," could never be more justly applied than in the present case. He taxes the druggists with the want of that which I am sorry to say, too many apothecaries are not possessed of, viz. Education. That there are illiterate druggists in many parts of the metropolis, as well as in other places, cannot be denied; but then, are there not many illiterate apothecaries also? I would ask the gentleman, what it is that makes druggists so much inferior? In point of education, one has an equal advantage with the other; and there are thousands who are capable of construing a Latin prescription, who who know not the smallest principle of the language. He concludes his epistle with these words: "If accuracy of judgment, genuineness of medicines, &c. be of any importance in the preparation of medical prescriptions, it scarcely needs be mentioned where they are most likely to be combined." Whatever may be his real sentiments of the accuracy of judgment required in compounding medicines, I would, contrary to his insinuation, venture to assert, that from the superior chemical knowledge possessed by most druggists, they are in this case to be preferred. Indeed, the advantages derived by the druggist from his superiority in chemical knowledge, are very numerous; it enables him to know with certainty what effects certain combinations will produce; and as substances, simple in themselves, are known to act upon each other in such manner as to produce the most dangerous poisons, it is obvious that those who are best acquainted with these principles, are the properest to be applied to. His knowledge also of the quality of drugs, and the methods with which he is well acquainted, of detecting any adulteration, is without doubt of the greatest use in discovering the genuineness of medicines; and how many apothecaries there are, who, from a deficiency in this respect, are incapable of distinguishing a good drug from a bad one.

Being impressed with these considerations on reading the very partial letter of Observator, I could not resist the inclination of committing them to paper; and if you will be kind enough to insert them in your useful Miscellany, you will much oblige,

Yours, &c.

A CONSTANT READER.

*January* 13, 1810.

---

*** Though the subject of the above, and Observator's letter, may not appear scientific, yet they are connected with another which we wish to see canvassed on the most enlarged and liberal scale; we mean, that of Medical Reform. It cannot be questioned, that apothecaries and druggists have both acted out of their proper sphere. The apothecaries are become physicians, and the druggists apothecaries. The consequence is, that with most of the rising, and even the passing generations, each branch has been educated with a view to the stations they expect to fill. But as this is not universally the case, both our Correspondents may be right as to individuals. The question, however, has quite a different bearing. Whilst the fees of physicians remain as at present, an order of medical men is absolutely necessary to visit the patient frequently in all acute, perhaps many chronic, cases; and the only established mode of payment is by their profit on the medicines. This is only one part of the question unnoticed by these gentlemen; there are many others which will occur to every reader, though our Correspondents, in their zeal or haste, have omitted to touch upon them. EDIT.

